# Crocin Ameliorates Atopic Dermatitis Symptoms by down Regulation of Th2 Response via Blocking of NF-κB/STAT6 Signaling Pathways in Mice

**DOI:** 10.3390/nu10111625

**Published:** 2018-11-02

**Authors:** Yoon-Young Sung, Ho Kyoung Kim

**Affiliations:** Herbal Medicine Research Division, Korea Institute of Oriental Medicine, 1672 Yuseong-daero, Yuseong-gu, Daejeon 34054, Korea; yysung@kiom.re.kr

**Keywords:** *Gardenia jasminoides*, eosinophils, NC/Nga, Immunoglobulin E, thymus and activation-regulated chemokine

## Abstract

Crocin, a major constituent of *Gardenia jasminoides*, is a natural colorant carotenoid compound that has been reported to have anti-inflammatory effects. This study investigated the therapeutic effects of crocin on mice with atopic dermatitis induced by *Dermatophagoides farinae* crude extract, which is a common environmental allergen in house dust that causes atopic dermatitis in humans. Crocin application ameliorated *Dermatophagoides farinae* crude extract-induced atopic dermatitis symptoms by inhibiting the dermatitis severity score, ear thickness, and serum immunoglobulin E levels in NC/Nga mice. The increases in epidermal thickness and dermal inflammatory cells (eosinophil and mast cells) infiltrations observed on the dorsal back skin of atopic dermatitis control mice were inhibited in a dose-dependent manner by topical application of crocin in atopic dermatitis treatment mice. Crocin inhibited the *Dermatophagoides farinae* crude extract-induced increase of thymus and activation-regulated chemokines, interleukin (IL)-4, and IL-13 on the dorsal skin of mice. Crocin also inhibited *Dermatophagoides farinae* crude extract-induced activation of nuclear factor-κB (NF-κB) and signal transducer and activator of transcription (STAT) 6. These results show that crocin ameliorates atopic dermatitis symptoms by down regulation of the Th2 cells-mediated immune response via blocking of NF-κB/STAT6 signaling pathways.

## 1. Introduction

Atopic dermatitis (AD) is a pruritic, chronic, skin inflammatory disease that includes a prevalent T helper (Th) 2-mediated immune reaction to sustained exposure of allergens [1]. AD affects 15–20% of children and 1–3% of adults worldwide, and its prevalence continues to increase in developing countries and low-income countries, leading to a significant reduction in quality of life and economic burden [2]. Development of AD in infancy and subsequent allergic rhinitis and asthma in later childhood is referred to as the atopic march [3]. This disease is usually characterized by predominant expression of Th2-type cytokines, including interleukin (IL)-4, IL-5, and IL-13, and is associated with elevated circulating immunoglobulin E (IgE) and eosinophilia [4,5]. The imbalance of Th2 to Th1 cytokines in AD induces alterations in cell-mediated immune responses and IgE-mediated hypersensitivity, both of which play pivotal roles in the development of AD [6]. In addition, several cell types, including T lymphocytes, Langerhans cells (DCs), eosinophils, and mast cells, have been observed to infiltrate both the dermis and epidermis of patients with AD [7,8]. The development and pathophysiology of AD is complex and multifactorial because it involves dysfunction of the skin barrier, altered immune function, genetic factors, and environmental factors [9]. AD therapeutic strategies have been dominated by the application of local or systemic corticosteroids, however, the prolonged use of steroids often produces adverse effects in patients with AD [10]. Topical calcineurin inhibitors (tacrolimus and pimecrolimus) have become a popular treatment option for AD, however, there is a possible risk of lymphoma associated with the use of calcineurin inhibitors [11]. Therefore, there is a great need to develop new and effective AD therapies.

Crocin is a natural colorant carotenoid compound that is a major constituent of *Gardenia jasminoides*, which has been widely used for its homeostatic, antiphlogistic, analgesic, and antipyretic effects [12]. Our previous study indicated that the *Gardenia* fruit has anti-inflammatory and anti-allergic effects on house dust mite-induced AD mice and mast cells [13]. Crocin has multiple effects including anti-oxidant, anti-cancer, anti-inflammatory, and anti-atherosclerotic effects in various cell types [14]. It was recently reported that crocin ameliorates allergic airway inflammation by modulating IL-4/IL-13 or mitogen-activated protein kinase (MAPK) signaling in a murine asthma model [15,16]. Crocin also suppresses Th2 chemokines, such as thymus and activation-regulated chemokine (TARC/CCL17) and macrophage-derived chemokine (MDC/CCL22), in tumor necrosis factor (TNF)-α/interferon (IFN)-γ-stimulated human epidermal keratinocytes, known as HaCaT cells, which highlight the potential of crocin as an anti-atopic drug [17]. Although studies on the anti-inflammatory effects of crocin have been performed, the effects of crocin on reducing skin inflammation reactions and improving AD symptoms have not yet been evaluated. Thus, it is reasonable to propose that crocin may ameliorate AD through reducing the allergic immune response.

To test this hypothesis, we treated NC/Nga mice with *Dermatophagoides farinae* crude extract (DfE), a common environmental allergen in house dust that causes AD in humans, to induce human AD-like skin lesions, and assessed the inhibitory effect of crocin on the development of AD [18]. The effects of crocin were compared with those of tacrolimus, an immunosuppressant commonly used to AD. 

## 2. Materials and Methods

### 2.1. Drugs and Reagents

**Figure 1:**
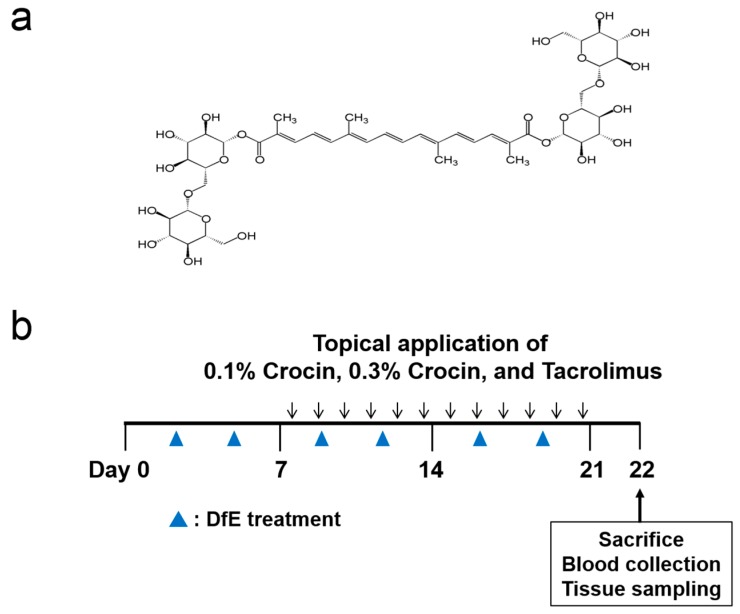
(**a**) Chemical structure of crocin and (**b**) experimental scheme for induction of atopic dermatitis (AD) and treatment of drugs in NC/Nga mice.

Crocin was purchased from Sigma Aldrich Co. (St. Louis, MO, USA) (Figure 1a). Protopic (Tacrolimus ointment 0.1%) was purchased from Astellas Pharma Inc. (Deerfield, IL, USA).

### 2.2. Animals

NC/Nga male mice (23–28 g, 6 weeks of age) were purchased from Central Lab. Inc. (Seoul, Korea) and maintained at Laboratory Animal Research Center of Korea Institute of Oriental Medicine (KIOM, Daejeon, Korea). Mice were housed in a specific pathogen-free conditions room with a 12-h light/dark cycle at 22 ± 1 °C and 50 ± 10% humidity. Mice were given access to a standard laboratory diet and water ad libitum. After acclimating for 2 weeks, the mice were randomly divided into five groups. All animal procedures were conducted in accordance with National Institutes of Health (NIH) Guidelines (NIH publication number 85-23. Revised 1996) and were approved by the Institutional Animal Care and Use Committee of the KIOM (Approval Number 17-024). 

### 2.3. Induction of AD and Treatment of Drug in Mice

Atopic dermatitis-like skin lesions in mice were induced using the procedure described previously [19]. The experimental schedule is provided in Figure 1b. The hair on the upper back of each mouse was removed and no further treatment was initiated for 24 h. Next, 4% sodium dodecyl sulfate (150 μL) was applied to the shaved dorsal skin and both surfaces of each ear (except the six mice in the Normal group) to disrupt the skin barrier. After 3 h, 100 mg of DfE ointment (Biostir Inc., Kobe, Japan) was applied to the skin. The DfE ointment treatment was repeated two times a week for 3 weeks. The Normal group consisted of six mice that received no treatment. The remaining mice were then assigned into one of four additional groups to explore the effects of crocin and tacrolimus (TAC) as follows: Control group, application of 100 mg DfE ointment only (6 mice); 0.1% Crocin group, application of 100 mg 0.1% Crocin (6 mice); 0.3% Crocin group, application of 100 mg 0.3% Crocin (6 mice); and TAC group, application of 100 mg TAC as a positive control (6 mice).

Crocin was dissolved in vehicle (70% ethanol) solution. The same volume of vehicle was applied to the normal and control groups. Seven days after the first DfE application, crocin or TAC were applied to the skin daily for 14 days. Mice were sacrificed on Day 22, and blood was collected from the inferior vena cava. The dorsal skin and ear tissues of mice were excised and subjected to histological examination.

### 2.4. Evaluation of Ear Thickness and Dermatitis Severity

Ear thickness was measured once each week using a micrometer (Mitutoyo Corporation, Kanagawa, Japan). The severity of dermatitis on the ear and dorsal skin lesions was evaluated twice each week. The development of erythema/hemorrhage, scarring/dryness, edema, and excoriation/erosion was scored as 0 (no symptoms), 1 (mild), 2 (moderate), and 3 (severe). The clinical dermatitis severity score for each mouse was defined as the sum of the individual scores using the method described in [20]. 

### 2.5. Measurement of Serum IgE Levels

Blood samples from the mice were centrifuged at 2000× *g* for 20 min at 4 °C, and then serum was collected and stored at −70 °C for further investigations. Total IgE concentration in the serum was measured using a mouse total IgE enzyme-linked immunosorbent assay (ELISA) kit (Shibayagi, Gunma, Japan) according to the manufacturer’s instructions.

### 2.6. Histopathological Analysis

Samples from ear and dorsal back skin tissues of the mice were separated and fixed in 10% neutral buffered formalin, embedded in paraffin, and then sliced into 3–4-μm serial sections. Representative sections were stained with hematoxylin and eosin (H&E) for general histopathology, Congo red (CR) stain for eosinophils, and toluidine blue (TB) stain for mast cells according to previously established methods [21,22]. The histological profiles of individual sections of the ear and dorsal skin were observed under a light microscope (Model Eclipse 80i, Nikon, Tokyo, Japan). To describe the changes in more detail, total thickness of the ear including cartilage (μm), dorsal skin epidermal thicknesses (μm), and numbers of inflammatory cells infiltrated in the dorsal skin dermis (cells/mm^2^ of dermis) were calculated using H&E stained tissue samples and a computer-assisted image analysis program (iSolution FL ver 9.1, IMT i-solution Inc., Vancouver, QC, Canada). In addition, mean numbers of eosinophils in CR-stained dorsal skin dermis samples (cells/mm^2^ of dermis) and mean numbers of mast cells in TB-stained back skin dermis samples (cells/mm^2^ of dermis) were determined using a computer-assisted image analysis program according to previous methods [23,24] with some modifications. At least five histological fields per each section from each mouse were considered to calculate each mean histomorphometrical value.

### 2.7. Contents of Cytokines and Chemokine in Skin Tissue

Contents of TARC, IL-4, IL-5, IL-13, IFN-γ, and IL-12 from the dorsal skin of mice were measured by ELISA using commercially available kits (R&D systems, Minneapolis, MN, USA). Approximately 10–15-mg tissue samples were homogenized in a homogenizer containing Pro-prep protein extraction solution (Intron, Seoul, Korea). Each sample was run in duplicate, and the BCA (bicinchoninic acid) Protein Assay Kit (Thermo Scientific, Rockford, IL, USA) was used for quantitative assessment of total protein content. Data are expressed as pg/mg of protein.

### 2.8. Preparation of Total Cell Lysates and Nuclear Extracts and Western Blotting

Extraction of total cell lysates from the back skin tissue samples of mice was performed using PRO-PREP protein extraction solution (Intron). Proteins (15 μg) were separated by 10% sodium dodecyl sulfate-polyacrylamide gel electrophoresis and transferred onto nitrocellulose membranes. Membranes were blocked with 5% bovine serum albumin and then incubated with primary antibodies against IκBα, phospho-IκBα, signal transducer and activator of transcription (STAT) 6, phospho-STAT6, and β-actin (Cell Signaling Technology, Beverly, MA, USA) at 4 °C overnight. The membranes were then incubated with the corresponding horseradish peroxidase-conjugated secondary antibodies (Cell Signaling) for 1 h at room temperature. Membranes were treated with the enhanced chemiluminescence (ECL) detection reagent (Amersham Bioscience, Buckinghamshire, UK), and protein bands were visualized using the Fujifilm Luminescent Image Analyzer Las-1000 (Fujifilm, Tokyo, Japan).

### 2.9. Statistics

Results are expressed as mean ± standard error of the mean (S.E.M.). Statistical significance was determined using one-way analysis of variance (ANOVA) followed by multiple comparison test. Differences was considered significant at *p* < 0.05.

## 3. Results

### 3.1. Effects of Crocin on AD-Induced NC/Nga Mice

**Figure 2:**
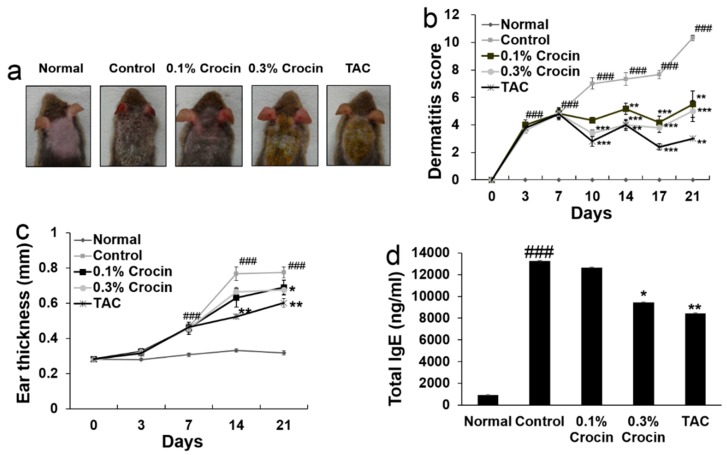
(**a**) Clinical features; (**b**) dermatitis severity score; (**c**) ear thickness; and (**d**) total immunoglobulin E (IgE) levels in serum. Results are expressed as mean ± standard error of the mean (S.E.M.) for six mice. Groups: Normal, no treatment group; Control, DfE-treated group; Crocin, 0.1%- or 0.3%-treated group; and TAC, tacrolimus-treated group. Key: ^###^ *p* < 0.001 versus Normal group; * *p* < 0.05, ** *p* < 0.01, and *** *p* < 0.001 versus Control group.

To investigate the effect of crocin treatment on AD-like symptoms, we evaluated the effects of crocin on DfE-induced AD model mice (NC/Nga) using clinical features, including dermatitis severity score and ear thickness. As shown in Figure 2a, repeated application of DfE for 21 days induced AD-like symptoms such as dryness, erythema, hemorrhage, edema, scarring, erosion, and excoriation. These AD symptoms were alleviated in mice treated with skin applications of 0.1% crocin, 0.3% crocin, or TAC. Dermatitis severity scores increased rapidly in the DfE-induced control mice and were significantly ameliorated in mice in the 0.1% Crocin, 0.3% Crocin, or TAC treatment groups (Figure 2b). Ear thickness increased in the DfE-induced control mice, and the increased ear thickness was significantly reduced by application of 0.3% crocin or TAC (Figure 2c). These results show that crocin ameliorates DfE-induced AD symptoms in NC/Nga mice.

### 3.2. Effect of Crocin on Serum IgE Levels

To investigate whether crocin treatment inhibited the increased serum IgE levels that are a hallmark of AD, we examined serum levels of IgE in the DfE-induced NC/Nga mice after crocin application. The total IgE levels in the serum were significantly decreased in the 0.3% crocin group (Figure 2d).

### 3.3. Effects of Crocin on Histological Features

**Figure 3:**
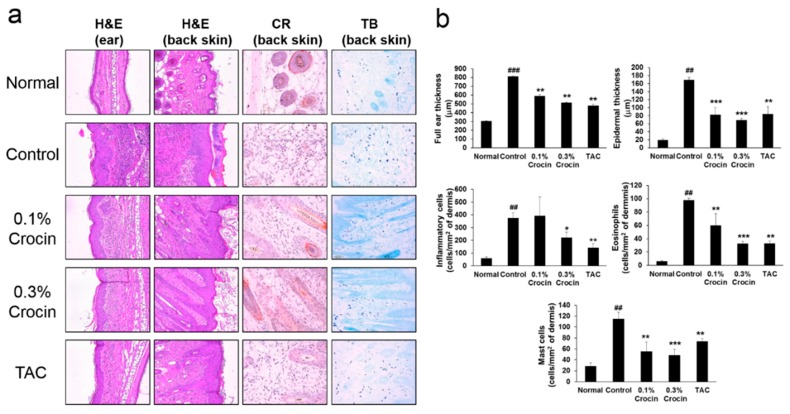
Histopathology and histomorphometrical analysis of the ear and back skin tissues. (**a**) Ear and back skin sections were stained with hematoxylin and eosin (H&E; original magnification ×200). Back skin sections were stained with Congo red (CR; ×400) or toluidine blue (TB; ×200). (**b**) Full ear thickness in the ear, epidermal thickness, inflammatory cells number, eosinophils number, and mast cells number in the back skin. Results are expressed as mean ± standard error of the mean (S.E.M.) for six mice. Groups: Normal, no treatment group; Control, DfE-treated group; Crocin, 0.1%- or 0.3%-treated group; TAC, tacrolimus-treated group. Key: ^##^ *p* < 0.01 and ^###^ *p* < 0.001 versus Normal group; * *p* < 0.05, ** *p* < 0.01, and *** *p* < 0.001 versus Control group.

The histopathological features of the ear and dorsal skin lesions in the mice are shown in Figure 3. We observed increased ear and epidermal thickness, inflammation of the dermis, and infiltration of eosinophil and mast cells into the ear and dorsal back skin of AD control NC/Nga mice, relative to normal mice. These results suggested classic AD histopathological findings. However, these DfE-induced AD-related histopathological signs on the ear and dorsal back skin samples were simultaneously and significantly (*p* < 0.05) inhibited by application of crocin in a dose-dependent manner as compared to those of AD control. In particular, we found that treatment of mice with 0.3% crocin showed comparable inhibitory activities as TAC on the DfE-induced AD ear and dorsal skin lesions.

### 3.4. Effects of Crocin on Protein Contents of Chemokine and Cytokines in the Dorsal Skin

To investigate whether crocin treatment could alter the immune response, we assessed the effect of crocin on protein levels of chemokine and cytokines in the dorsal skin lesions of the mice (Table 1). We observed an increase of Th2 chemokine (TARC) and Th2 cytokines (IL-4, IL-5, and IL-13), and also saw a decrease in Th1 cytokines (IFN-γ and IL-12) protein expression levels in DfE-induced AD control mice. However, crocin treatment inhibited the DfE-induced upregulation of TARC, IL-4, and IL-13. Crocin treatment did not change protein expression of Th1 cytokines. These results indicate that crocin ameliorates AD symptoms by downregulating the Th2 immune response in the DfE-treated NC/Nga mice.

### 3.5. Effects of Crocin on Activation of NF-κB and STAT6 in the Back Skin

**Figure 4:**
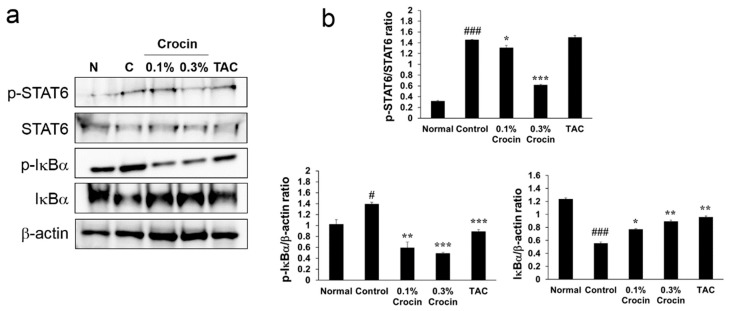
Effects of crocin on *Dermatophagoides farinae* extract (DfE)-induced NF-κB and signal transducer and activator of transcription 6 (STAT6) activation in the back skin tissues. Expression of IκBα, p-IκBα, p-STAT6, STAT6, and β-actin protein were evaluated by measuring protein levels in the total extracts using Western blotting (**a**). The relative abundance of proteins was calculated for the p-STAT6/STAT6, p-IκBα/β-actin, and IκBα/β-actin ratios (**b**). Values represent the mean ± standard error of the mean (S.E.M) of three independent experiments. ^#^ *p* < 0.05 and ^###^ *p* < 0.001 versus Normal group; * *p* < 0.05, ** *p* < 0.01 and *** *p* < 0.001 versus Control group.

To investigate the regulatory mechanisms of crocin treatment in AD, we assessed the effect of crocin on the activation of nuclear factor (NF)-κB and STAT6 in the dorsal skin lesions of DfE-treated NC/Nga mice (Figure 4). We found that crocin treatment inhibited DfE-induced phosphorylation of STAT6 in a dose-dependent manner. However, TAC did not significantly inhibit the phosphorylation of STAT6. In addition, crocin inhibited DfE-induced phosphorylation and degradation of IκBα (signaling molecule leading to NF-κB activation) in a dose-dependent manner. These results suggest that crocin inhibits production of DfE-induced chemokines/cytokines by suppressing the activation of both STAT6 and NF-κB.

## 4. Discussion

*Gardenia jasminoides* has been used in traditional medicine for treatment of inflammation, edema, and dermatitis [11]. Crocin, which is a natural carotenoid derived from *Gardenia jasminoides*, has anti-inflammatory effects [14,15,16,17]. Nevertheless, there have been no reports of its possible anti-AD effects. This study evaluated the therapeutic effects of topical crocin application to NC/Nga mice that were exposed to house dust mite allergen. We found that crocin treatment effectively attenuated DfE-induced AD-like symptoms by reducing the dermatitis severity score, ear thickness, IgE levels in serum, and infiltration of inflammatory cells, including eosinophils and mast cells, in NC/Nga mice. We also found that crocin treatment lowered levels of the chemokine TARC and Th2 cytokines (IL-4 and IL-13) in DfE-treated NC/Nga mice. These findings suggest that topical application of crocin might provide a novel therapeutic regimen for the treatment of various skin diseases, including AD.

In this study, we found that elevated Th2-associated factors IL-4, IL-13, and TARC levels in the dorsal skin lesions caused by a mite allergen were decreased by crocin treatment in mice. The pathophysiology of AD involves dysregulated Th1 and Th2 responses, which are characterized by Th2-dominant allergic inflammation mediated by IL-4, IL-5, and IL-13 [25]. IL-4 and IL-13 activate mast cells and basophils by regulating Th2 differentiation and IgE production by B cells, whereas IL-5 is involved in eosinophil development, survival, and proliferation in AD [1]. Elevated levels of Th2 cytokines are detected in the skin of AD patients, and these elevated levels correlate with increased circulating IgE levels [26]. The Th2 chemokine family includes TARC/CCL17, which binds to CC chemokine receptor 4-positive (CCR4) cells. Th2 cells are attracted by the TARC produced in epidermal keratinocyte or epidermal dendritic cells [27]. In turn, Th2-mediated cytokines (IL-4, IL-5, and IL-13) secondarily stimulate keratinocytes, which leads to increased production of TARC [28]. Abnormally high levels of TARC in serum of AD patients indicate accelerated pathogenesis of cutaneous inflammation [29], thus we reason that normalization of TARC level using appropriate topical treatment may alleviate accelerated inflammation [30]. These data indicate that crocin treatment suppressed DfE-induced AD symptoms in NC/Nga model mice by downregulating the immune response mediated by Th2 cells. 

To further elucidate the mechanism underlying the anti-AD effects of crocin, we investigated NF-κB and STAT6 activation in the back skin. NF-κB is a major transcription factor that is important in the development of acute and chronic inflammatory diseases [31]. In addition, NF-κB signaling pathways are reportedly involved in the production of cytokines and chemokine in human keratinocytes, including HaCaT cells [32,33]. A previous study showed that crocin treatment suppressed Th2 chemokines (TARC and MDC) by inhibiting extracellular signal-regulated kinase-MAPK/NF-κB/STAT 1 signaling pathways in TNF-γ/IFN-γ-stimulated human keratinocytes [17]. Therefore, we focused on chemokines and inflammatory cytokines released from AD-like skin lesions and their mechanisms in vivo. In this study, we found that crocin treatment improved DfE-induced AD-like lesions through suppression of chemokine and inflammatory cytokine expressions via inhibition of NF-κB activation (phosphorylation and degradation of IκBα) and STAT6 phosphorylation in the back skin of mice. In AD, STAT6 is a critical transcriptional factor that regulates IL-4-mediated immune responses. STAT-6 is phosphorylated and activated via an IL-4 receptor-mediated signal and subsequently regulates IL-4-mediated transcriptional events, including Th2 differentiation, expression of cell surface markers, and Ig class switching to IgE [34]. These data indicate that crocin suppressed Th2-related cytokines (IL-4 and IL-13) and Th2 chemokine TARC by blocking NF-κB and STAT6 signaling pathways in the DfE-induced AD mice. Thus, crocin may be an effective alternative candidate for the treatment of AD. 

## 5. Conclusions

This study showed that the topical application of crocin on NC/Nga mice with DfE-induced AD effectively ameliorated the development of AD-like skin lesions. The mechanism of this effect appears to be suppression of the Th2 cells-mediated immune response via blockage of NF-κB/STAT6 signaling pathways. In summary, crocin ameliorated DfE-induced AD-like symptoms through suppressing NF-κB and STAT6 signaling pathways.

## Figures and Tables

**Table 1:** Protein contents of chemokine and cytokines in the dorsal skin lesions.

	Normal	Control	0.1% Crocin	0.3% Crocin	TAC
TARC (pg/mg)	4.78 ± 0.64	8.22 ± 0.80 ^#^	9.61 ± 1.70	2.10 ± 0.35 *	2.79 ± 0.86 **
IL-4 (pg/mg)	2.00 ± 0.64	4.77 ± 0.65 ^#^	1.62 ± 0.40 **	0.98 ± 0.05 **	1.96 ± 0.45 *
IL-5 (pg/mg)	2.35 ± 0.41	3.88 ± 0.46	2.78 ± 0.22	3.11 ± 0.33	4.40 ± 0.66
IL-13 (pg/mg)	2.24 ± 0.47	5.81 ± 1.52 ^#^	1.55 ± 0.17 *	1.00 ± 0.13 *	2.02 ± 0.14 *
IFN-γ (pg/mg)	11.57 ± 1.94	4.88 ± 0.39 ^#^	5.34 ± 0.85	5.43 ± 0.51	6.36 ± 0.34 *
IL-12 (pg/mg)	1.45 ± 0.26	0.49 ± 0.09 ^#^	0.97 ± 0.25	0.76 ± 0.10	0.91 ± 0.09 *

Values are expressed as means ± S.E.M. (*n* = 6). Key: ^#^ *p* < 0.05 compared with the Normal group; * *p* < 0.05, ** *p* < 0.01 compared with the AD control group. TARC, thymus and activation-regulated chemokine; TAC, tacrolimus; IL, interleukin.
